# The identification and differentiation of the *Candida parapsilosis* complex species by polymerase chain reaction-restriction fragment length polymorphism of the internal transcribed spacer region of the rDNA

**DOI:** 10.1590/0074-02760150466

**Published:** 2016-04

**Authors:** Leonardo Silva Barbedo, Maria Helena Galdino Figueiredo-Carvalho, Mauro de Medeiros Muniz, Rosely Maria Zancopé-Oliveira

**Affiliations:** Fundação Oswaldo Cruz, Instituto Nacional de Infectologia Evandro Chagas, Laboratório de Micologia, Rio de Janeiro, RJ, Brasil

**Keywords:** Candida parapsilosis complex, PCR-RFLP, fungaemia

## Abstract

Currently, it is accepted that there are three species that were formerly grouped under *Candida parapsilosis*: *C. para- psilosis sensu stricto*, *Candida orthopsilosis*, and*Candida metapsilosis*. In fact, the antifungal susceptibility profiles and distinct virulence attributes demonstrate the differences in these nosocomial pathogens. An accurate, fast, and economical identification of fungal species has been the main goal in mycology. In the present study, we searched sequences that were available in the GenBank database in order to identify the complete sequence for the internal transcribed spacer (ITS)1-5.8S-ITS2 region, which is comprised of the forward and reverse primers ITS1 and ITS4. Subsequently, an *in silico* polymerase chain reaction-restriction fragment length polymorphism (PCR-RFLP) was performed to differentiate the *C. parapsilosis* complex species. Ninety-eight clinical isolates from patients with fungaemia were submitted for analysis, where 59 isolates were identified as *C. parapsilosis sensu stricto*, 37 were identified as *C. orthopsilosis*, and two were identified as *C. metapsilosis*. PCR-RFLP quickly and accurately identified *C. parapsilosis* complex species, making this method an alternative and routine identification system for use in clinical mycology laboratories.

The incidence of fungaemia, i.e., candidaemia, keeps increasing steadily (Aittakorpi et al. 2012), especially in hospitalised immunocompromised patients (Takuma et al. 2015). While *Candida* spp are the third or fourth most common causative agents of fungaemia (Cantey & Milstone 2015),*Candida parapsilosis* oscillates between the second and fourth most common agent of candidaemia in hospitals throughout the United States of America (Wisplinghoff et al. 2014), Europe (Cobos-Trigueros et al. 2014), Asia (Wu et al. 2014), and Latin America (Nucci et al. 2013). *C. parapsilosis* has been associated with either localised or deep-seated infections (Trofa et al. 2008). Candidaemia caused by *C. parapsilosis* is generally related to the presence of a central venous catheter (Hu et al. 2014), as well as the use of parenteral nutrition (Hirai et al. 2014), and *C. parapsilosis* is the predominant species that causes bloodstream infections in premature newborns in neonatal intensive unit care (Pammi et al. 2014).

Currently, it is accepted that there are three species that were formerly grouped under*C. parapsilosis*: *C. parapsilosis sensu stricto*,*Candida orthopsilosis*, and *Candida metapsilosis*(Tavanti et al. 2005). In fact, characteristics such as the antifungal susceptibility profile (Bonfietti et al. 2012, Ruiz et al. 2013, Figueiredo-Carvalho et al. 2014), virulence attributes, as shown in human oral and epidermal tissues models (Gácser et al. 2007), the ability to produce extracellular proteases, lipase secretion, pseudohyphae formation (Németh et al. 2013), differences in biofilm biomass (Lattif et al. 2010), and the ability to cause tissue damage in the nonconventional host *Galleria mellonella* (Gago et al. 2014) have demonstrated the diversity of these species, including isolates recovered from clinically healthy animals (dogs, psittacines, raptors, and a prawn) (Brilhante et al. 2014).

Previous studies have shown that *C. metapsilosis* was associated with a reduced virulence as compared to *C. orthopsilosis* and *C. parapsilosis sensu stricto* (Orsi et al. 2010). This finding may reflect the decreased ability of *C. metapsilosis* to adhere to epithelial cells (Bertini et al. 2013). In addition, Constante et al. (2014) have shown that patients with candidaemia caused by *C. orthopsilosis* presented with different predisposing conditions to infection as compared to those infected by *C. parapsilosis sensu stricto*.

An accurate, faster, and economical identification of fungal species has been the main goal in mycology (Alnuaimi et al. 2014), especially when species complexes are involved because identification based on solely phenotypic characteristics is often inconclusive due the variability within the*C. parapsilosis* complex species. Molecular analyses have been used for this purpose, and a single step polymerase chain reaction (PCR) using a pair of universal primers [internal transcribed spacer (ITS)1 and ITS4] to amplify the ITS1-5.8S-ITS2 of the ribosomal DNA (rDNA) (genes encoding for ribosomal RNA) region has been considered as a barcoding sequence, i.e., the most widely used genetic marker in identifying species (Schoch et al. 2012). Therefore, the aim of this study was develop a new and reliable identification strategy to differentiate between the clinical *C. parapsilosis complex* isolates. We developed this strategy in order to correctly identify the species using a PCR associated with double enzymatic digestion [restriction fragment length polymorphism (RFLP)] and compared this method to the analysis of the partial D1/D2 region of the 28S rDNA gene sequences (Barbedo et al. 2015).

The reference strains of *C. parapsilosis*, *C. ortho- psilosis*, and *C. metapsilosis* that were available in the GenBank database were analysed to identify the complete sequences for the ITS1-5.8S-ITS2 region of the ATCC 22019 strain (*C. parapsilosis*, from Puerto Rico), the ATCC 96141 strain (*C. orthopsilosis*, from San Antonio, Texas, USA), and the ATCC 96143 strain (*C. metapsilosis*, from Livermore, California, USA) under the GenBank accessions AY939798, EU564208, and EU564207, respectively. These regions comprised the forward and reverse primers ITS1 (5’-TCCGTAGGTGAACCTGCGG-3’) and ITS4 (5’-TCCTCCGCTTATTGATATGC-3’). *In silico* PCR amplification using the FastPCR v.6.0 software was performed using the ITS1 and ITS4 primers. The selected sequences were used to determine the length of the products and *in silico* RFLP was performed using the pDRAW32 DNA analysis software v.1.1.125 database. *Hha*I and *Sau96*I were selected based on the presence of cleavage sites that generated fragments that could discriminate between the species complex on a conventional agarose gel. Species-specific variations were thus identified according to the restriction enzyme banding profile. Double digestion with*Hha*I and *Sau96*I cut the ITS1-5.8S-ITS2 PCR products of the *C. parapsilosis* complex reference strains in three different molecular patterns, producing 117, 178, and 225 bp fragments for ATCC 22019 (*C. parapsilosis*), 102, 183, and 225 bp fragments for ATCC 96141 (*C. orthopsilosis*), and 114, 187, and 228 bp fragments for ATCC 96143 (*C. metapsilosis*) (Supplementary Figure).

Ninety-eight clinical strains of *C. parapsilosis lato sensu* that were isolated from the bloodstream and catheter of critically ill patients and that were maintained at the Fungal Culture Collection of Evandro Chagas National Institute of Infectious Diseases/Oswaldo Cruz Foundation, Brazil were included in this study, which was approved by the Ethical Committee of the same institution. Information about all isolates (except isolates 83 and 84), including species identification, microsatellite typing of *C. para- psilosis sensu stricto*, and the sequences of the D1/D2 region of the 28S rDNA gene, have previously been published (Barbedo et al. 2015).

Yeast cells were grown on Sabouraud dextrose agar (Difco, USA) and genomic DNA was extracted using the Gentra^®^ Puregene^®^ Yeast/Bact. Kit (Qiagen^®^, USA) according to the manufacturer’s protocol. The DNA concentration was determined with a spectrophotometer (NanoVue Plus^TM^; GE Healthcare, USA). The sample was run on a 1% agarose gel at 90 V for 80 min using gel electrophoresis and the gel was stained with ethidium bromide (0.5 µg mL^-1^) (Sigma-Aldrich, USA). The integrity of the DNA was analysed under ultraviolet light. DNA was stored at -20ºC until future use.

PCR analysis of the ITS1-5.8S-ITS2 region of the rDNA gene was performed using a final volume of 50 µL. Each reaction mixture contained 50 ng of DNA, 1X PCR buffer [10 mM Tris-HCl (pH 8.4), 50 mM KCl, 1.5 mM MgCl_2_ (Invitrogen^TM^ Brazil)], 0.2 mM (each dNTP) dATP, dCTP, dGTP, and dTTP (Invitrogen^TM^, USA), 2.5 U recombinant DNA polymerase (Invitrogen^TM^, Brazil), and 50 ng of each of the forward (ITS1) and reverse (ITS4) primers. The PCR was performed in a Bio-Rad model C 1000 using the following program: initial denaturation at 95ºC for 5 min, followed by 30 cycles of denaturation for 1 min at 95ºC, annealing for 1 min at 55ºC, extension for 1 min at 72ºC, and a final extension step at 72ºC for 5 min. The PCR products (25 µL) were double digested with *Sau96*I (10 U/µL) and *Hha*I (20 U/µL) for 3 h at 37ºC, and the digested products were mixed with 0.2 volumes of loading buffer and were separated on a 3% agarose gel at 100 V using gel electrophoresis for approximately 2 h. After staining with ethidium bromide (0.5 µg mL^-1^), the RFLP patterns were assigned visually based on the fragments obtained on the ATCC strains banding profiles on electrophoresis.

All 98 clinical isolates were identified molecularly using PCR-RFLP, and 59 isolates were identified as *C. parapsilosis sensu stricto*, 37 were identified as*C. orthopsilosis*, and two were identified as *C. metapsilosis* (Figure). These results are in agreement with the DNA sequencing results of the D1/D2 region of the 28S rDNA gene that were previously described (Barbedo et al. 2015).

**Figure:**
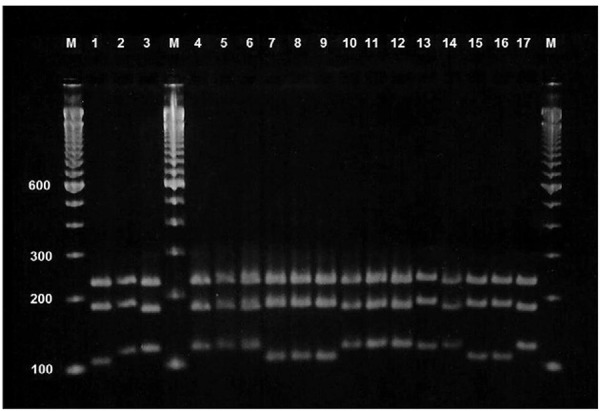
Representative agarose (3%) gel electrophoresis (at 100 V for approximately 2 h) of restriction digestion of internal transcribed spacer (ITS)1-5.8S-ITS2 region of the ribosomal DNA amplicons (with the primers ITS1 and ITS4) with *Hha*I and *Sau96*I from reference strains and 14 selected *Candida parapsilosis sensu lato* strains. Lane M: 100 bp DNA ladder; 1: *Candida orthopsilosis* ATCC 96141; 2: *Candida metapsilosis* ATCC 96143; 3: *C. parapsilosis sensu stricto* ATCC 22019. The restriction fragment length polymorphism patterns in lanes 4-6, 10-12, 14, and 17 were classified as*C. parapsilosis sensu stricto*; 7-9, 15, and 16 were classified as *C. orthopsilosis*, whilst in 13 was classified as *C. metapsilosis*. The positions of migration of the fragments 100, 200, 300, and 600 bp are indicated.

The emergence of new species of *Candida* as potential pathogens is a reflection of changing scenarios in medicine since the 1960s (Giri & Kindo 2012). *C. parapsilosis* emerged in recent decades across the globe as an important nosocomial pathogen in invasive fungal infections with haematogenous dissemination (Costa et al. 2014). Since 2005, based on multilocus sequence typing, the pathogen has been considered to be a *C. parapsilosis* complex (Tavanti et al. 2005). Morphological and biochemical identification methods are time-consuming, require trained experts, and, in most cases, do not differentiate between the species involved in the complexes (Irinyi et al. 2015).

Alternatively, various molecular methodologies, including PCR-RFLP, have been used for rapid identification, offering a practical approach to identifying species that are most demanding in terms of taxonomic expertise. PCR-RFLP of partial regions of different genes (*SADH*, *IGS1*, and *FKS1*) with only one restriction enzyme (*Ban*I, *Nla*III,*Rsa*I, *Hinf*I, and *EcoR*I) has been described to be able to differentiate between the *C. parapsilosis*complex species (Asadzadeh et al. 2009, Mohammadi et al. 2015). However, there were contradictory results regarding two strains on the amplification of a*FKS1* region followed by an *EcoR*I digestion, as well as a *SADH* region followed by *Bam*I digestion (Abi-Chacra et al. 2013).

Here, we describe the assessment of the different molecular patterns within the*C. parapsilosis* complex using only one-step PCR of the ITS1-5.8S- ITS2 rDNA region associated with the best choice of restriction enzymes according to*in silico* and in vitro analyses. The PCR-RFLP profiles were informative and generated distinct banding patterns for each species, allowing their differentiation. Double digestion with *Hha*I and *Sau96*I produced three fragments: *C. orthopsilosis* was better differentiated by the third fragment (102 bp), which was smaller in size compared to the fragments for*C. parapsilosis sensu stricto* and *C. metapsilosis*. On the other hand, the second fragment (187 bp) in *C. metapsilosis* is above the *C. parapsilosis sensu stricto* fragment. This proposed identification technique for comparing reference strains and clinical isolates is simple, reliable, faster, more affordable, and requires less technical expertise than sequencing.

Because of the heterogeneity in the ITS region of rDNA for each of the *C. parapsilosis* complex species, studies have also suggested high genetic variability among clinical *C. orthopsilosis* isolates compared to*C. parapsilosis sensu stricto* isolates, which are predominantly clonal and exhibit limited genotypic variations (Merseguel et al. 2015). Asadzadeh et al. (2015) identified three different haplotypes among 19 *C. orthopsilosis* isolates based on the DNA sequence data of the ITS region and the divergent nucleotides at the 58, 78, 79, 109, 142, 143, 144, 145, and 414 positions. However, this study found that *Sau96*I and *Hha*I double digestion (positions 102/103 and 285/286, respectively) did not cleave in the same divergent nucleotide position previously described within the ITS region, supporting the use of this method.

Different molecular methodologies have been used to identify other*Candida* yeasts species involved in complexes, e.g., *Candida glabrata*, *Candida bracarensis*, and *Candida nivariensis* (Enache-Angoulvant et al. 2011), *Candida haemulonii*, *C. haemulonii*var. *vulnera* and *Candida duobushaemuloniii* (Cendejas-Bueno et al. 2012), *Candida guilliermondii*, *Candida fermentati*, and *Candida carpophila* (Medeiros et al. 2007), and *Candida rugosa sensu stricto*, *Candida pseudorugosa*, *Candida neorugosa*, and *Candida mesorugosa* (Padovan et al. 2013). rDNA is a common target in PCR-based molecular methods to identify*Candida* at the species level. PCR techniques using primers that span highly variable sequences within ITS1 and ITS2 and the conserved regions of the 18S, 5.8S and 28S rDNA genes have been used to differentiate medically important*Candida* species (Alnuaimi et al. 2014).

In conclusion, PCR-RFLP for the ITS-rDNA region allowed us to identify *C. parapsilosis* complex species quickly and accurately. This method provides an alternative routine identification system for use in clinical mycology laboratories because only single pair of primers and simple equipment are necessary. There are five important points demonstrating the relevance of this work: (i) this method can be completed in one PCR step, (ii) the use of universal primers (ITS1-5.8S-ITS2) is a more affordable approach (iii) the target of an important sequence (considered to be a barcoding sequence), (iv) using restriction enzymes corroborated by *in silico* and in vitro analyses gives robustness in our study with 100% success in comparison with other assays, and (v) the assay is not time-consuming. This is a fairly important aspect especially for hospitalised patients, where speciation is becoming relevant for the early identification and appropriate antifungal therapy for these patients.
